# Die palliative Extubation nach ROSC im Rettungsdienst – eine Handlungsempfehlung für Notärzte

**DOI:** 10.1007/s00063-025-01271-y

**Published:** 2025-04-29

**Authors:** Christian Volberg, Katharina Politt, Sebastian Passon, Nils Heuser, Elena Hofacker, Hinnerk Wulf

**Affiliations:** 1 Klinik für Anästhesie und Intensivtherapie, Universitätsklinikum Marburg, Philipps-Universität Marburg, Baldingerstraße, 35043 Marburg, Deutschland; 2 Institut für angewandte und klinische Ethik im Gesundheitswesen, Dekanat Humanmedizin, Philipps-Universität Marburg, Baldingerstraße, 35043 Marburg, Deutschland

**Keywords:** Reanimation, Lebensende, Patientenwille, DNR, Medizinethik, Resuscitation, End-of-life care, Advance directive, DNR, Medical ethics

## Abstract

Im präklinischen Alltag wird eine Reanimation bei Kreislaufstillstand nach Eintreffen des Rettungsdiensts so schnell wie möglich begonnen, um die „No-flow“-Zeit, die Zeit, in der kein Blut zirkuliert und somit die Organe nicht mit Sauerstoff versorgt werden, so gering wie möglich zu halten. Oft besteht erst nach Einleitung der medizinischen Maßnahmen die Möglichkeit, die Angehörigen nach dem vorausverfügten oder mutmaßlichen Willen der Person, z. B. festgelegt durch eine Patientenverfügung, zu fragen. Ist in dieser Verfügung die Ablehnung lebensrettender Sofortmaßnahmen (DNR = „do not resuscitate“, deutsch: „nicht reanimieren“) festgelegt, ist dieser Patientenwunsch für die Behandlung bindend. Konnte jedoch in der Zwischenzeit die Rückkehr zu einem Spontankreislauf (ROSC) erzielt werden, kommt es für das betreuende Rettungsteam zu einem ethischen Dilemma und Patienten werden oftmals gegen ihren vorausverfügten Willen in ein Krankenhaus gebracht. Hierdurch resultiert dann eine nicht gewollte Übertherapie. In dem folgenden Artikel werden anhand dreier Fallbeispiele die ethischen und rechtlichen Aspekte der palliativen Extubation nach Rückkehr des Spontankreislaufs bei festgelegtem „DNR“-Status diskutiert.

## Abstract (en)

In the prehospital emergency setting, resuscitation following cardiac arrest is initiated as soon as possible after arrival of the emergency services in order to minimize the no-flow time, i.e., the period during which there is no blood circulation and therefore no oxygen supply to the organs. There is frequently no opportunity to ask relatives about the prespecified or presumed wishes of the patient, e.g., in the form of a living will, until after initiation of emergency medical interventions. If an advance directive stipulates “do not resuscitate” (DNR), then this wish is legally binding for treatment. However, if return of spontaneous circulation (ROSC) has been achieved in the meantime, the treating emergency team faces an ethical dilemma, and patients are often taken to hospital against their prespecified will. This leads to unwanted overtreatment. Based on three case reports, the following article discusses the ethical and legal aspects of palliative extubation after ROSC in patients with a predefined DNR status.

## Einleitung

Die kardiopulmonale Reanimation ist bei Herz-Kreislauf-Stillstand die einzige Maßnahme, um einem leblosen Menschen die Chance auf ein Weiterleben zu ermöglichen. Um dieses Ziel zu erreichen und das Outcome der Betroffenen nachhaltig zu verbessern, wird in der Notfallmedizin, Intensivmedizin und Kardiologie ein immenser Forschungsaufwand betrieben und internationale Leitlinien werden stetig überarbeitet. Durch diese Anstrengungen kann mittlerweile jede 10. Person einen außerklinischen Herz-Kreislauf-Stillstand überleben [1]. Als kritische Anmerkung muss man jedoch mit einbeziehen, dass eine autonome Teilhabe am Leben nicht immer gegeben ist und mitunter schwere neurologische Einschränkungen durch den hypoxischen Hirnschaden entstehen. Diese Einschränkungen sind jedoch von vielen Menschen nicht gewünscht, sodass sie sich mithilfe von Patientenverfügungen absichern möchten. In der Notfallsituation ist es zu Beginn einer Reanimation für den behandelnden Arzt mitunter schwierig zu ermitteln, ob der Patient der Wiederbelebung überhaupt zustimmen würde. Angehörige sind aufgrund des akuten Eintritts ebenfalls oft nicht vorbereitet und ihnen fällt vielleicht erst im Verlauf der Wiederbelebung auf, dass die Maßnahme überhaupt nicht vom Patienten gewollt ist oder eine Patientenverfügung existiert. Hat der Patient aber in der Zwischenzeit schon einen eigenen Kreislauf entwickelt („return of spontaneous circulation“, ROSC), befindet sich das Rettungsteam in einem Entscheidungskonflikt. Muss der Patient nun in ein Krankenhaus transportiert werden, da er ja lebt, oder ist es auch zulässig, dass man die Therapie vor Ort beendet und ein Sterben im häuslichen Umfeld ermöglicht?

Da es auf diese Frage bisher keine hinreichende Literatur gibt, haben wir uns mit dieser sehr schwierigen Fragestellung beschäftigt und betrachten das Problem aus verschiedenen Blickwinkeln. Anhand dreier Fallbeispiele leiten wir in das Thema ein, beleuchten den Hintergrund und klären neben den medizinischen auch die juristischen sowie ethischen Aspekte der Problematik. Abschließend geben wir eine praktische Empfehlung für diese besondere Konstellation, sodass der Leser eine Hilfestellung an der Hand hat, sollte sich eine ähnliche Situation im Einsatzgeschehen ergeben.

## Fallbeispiele

### Fallbeispiel 1

Rettungswache in einer eher ländlichen Region Deutschlands, die Alarmierung des Rettungsdienstes (Notarzteinsatzfahrzeug [NEF] und Rettungswagen [RTW]) erfolgt um 13.21 Uhr mit dem Einsatzstichwort „Bolusgeschehen“.

Trotz etwa 10-minütiger Anfahrt kommt es zu keiner weiteren Rückmeldung, da der RTW aufgrund einer langen Anfahrt auch erst ca. 2 min vor dem NEF eintrifft.

Beim Eintreffen des NEF-Teams um 13.33 Uhr wird ein älterer, am Boden liegender Patient vorgefunden, der eine deutliche Zyanose aufweist. Das Basismonitoring ist bereits durch die RTW-Besatzung angebracht, wobei die SpO_2_-Messung nicht ableitbar ist. Der Blutdruck ist auskultatorisch nicht messbar, die Karotispulse jedoch beidseits schwach palpabel. Die Pupillen sind beidseits mittelweit und träge lichtreagibel.

Von der Besatzung des RTW ist dem reglosen Patienten bereits eine Sauerstoffmaske mit einem hohen Fluss (18 l/min) aufgesetzt worden, weiterhin ist eine Intubation vorbereitet worden. Der Bolus kann von der Notärztin geborgen und die endotracheale Intubation durchgeführt werden.

Im weiteren Verlauf zeigt sich zunächst weiterhin ein bradykarder Sinusrhythmus, jedoch sind die Karotispulse im kurzzeitigen Verlauf, trotz vorangegangener Akrinor-Gabe (ratiopharm GmbH, Ulm), nicht mehr palpabel, sodass eine kardiopulmonale Reanimation nach den Leitlinien des European Resuscitation Councils (ERC) begonnen wird. Nach ungefähr 6 min stellt sich ein Spontankreislauf ein, im Elektrokardiogramm (EKG) zeigt sich ein bradykarder Sinusrhythmus und die zentralen Pulse sind wieder tastbar.

In diesem Moment sucht die Tochter des Patienten das Gespräch mit dem Rettungsteam und berichtet, dass eine Reanimation nicht im Sinne des Vaters gewesen sei.

In der jetzt durchgeführten Anamnese stellt sich heraus, dass der Patient seit über 10 Jahren an einer Alzheimer-Demenz erkrankt ist und seit 10 Jahren auch pflegebedürftig ist. Der Erkrankungsverlauf ist laut Aussagen der Angehörigen in den letzten 5 Jahren progredient. Seit 3 Jahren erkennt er niemanden mehr und ist auch zunehmend aggressiver geworden.

Eine gültige Patientenverfügung, die auch vorgelegt wird, bestätigt den von der Tochter vorgetragenen Patientenwillen, dass keine Wiederbelebung durchgeführt werden soll. Die Notärztin entscheidet sich deshalb zu einem „Callback“ mit dem diensthabenden leitenden Notarzt (LNA) und führt ein ausführliches Gespräch mit der Familie. Aufgrund des von der Familie vorgetragenen mutmaßlichen Patientenwillens, der durch die vorliegende Patientenverfügung gedeckt wird, entscheidet sich die Notärztin daraufhin, eine palliative Extubation durchzuführen.

Nach Diskonnektion des Beatmungsgeräts sowie Beendigung der kreislaufunterstützenden Medikamentengabe kommt es rasch zu einem erneuten Abfall von Herzfrequenz, Sauerstoffsättigung und Blutdruck. Im Weiteren deformieren sich die QRS-Komplexe und der Herzrhythmus wird deutlich unregelmäßiger. Der Patient verstirbt 93 min nach Alarmierung an der Einsatzstelle im Beisein seiner Angehörigen.

### Fallbeispiel 2

Alarm um 21.32 Uhr für das NEF einer eher ländlichen Rettungswache mit dem Einsatzstichwort „bewusstlose Person“.

Bereits auf der Anfahrt erfolgt die Rückmeldung durch den bereits eingetroffenen RTW einer nun begonnenen Reanimation, in der weiteren Rekonstruktion ergibt sich eine „No-flow“-Zeit von circa 10 min.

Bei Eintreffen des NEF-Teams an der Einsatzstelle in der Wohnung des 84-jährigen Patienten werden bereits initiale Maßnahmen des Advanced Life Support (ALS) durch die RTW-Besatzung durchgeführt, während die Ehefrau (ebenfalls um die 80 Jahre alt) aufgelöst im Nebenzimmer wartet.

Es wird die leitliniengerechte Therapie nach ALS-Algorithmus fortgeführt, der erste EKG-Rhythmus zeigt eine Asystolie. Die Pupillen des Patienten sind weit und nicht lichtreagibel. Es erfolgt die endotracheale Intubation, wonach bei der Auskultation über der Lunge beidseits Rasselgeräusche zu vernehmen sind.

Die Anlage des i.v.-Zugangs gestaltet sich schwierig, ist aber im zweiten Versuch erfolgreich, sodass die erste Gabe von Adrenalin in Minute 8 erfolgen kann. Nach Fortführung der Maßnahmen erzielt das Rettungsdienstteam in Minute 14 einen ROSC. Durch weitere fraktionierte Gaben von Adrenalin und Noradrenalin kann die Kreislaufsituation so weit stabilisiert werden, dass die Anlage eines 12-Kanal-EKG, eine Blutdruckmessung und eine weitere Anamnese mit der Ehefrau möglich sind. Im EKG zeigt sich ein Blockbild über allen Ableitungen, wobei jedoch unklar ist, ob dieses neu aufgetreten oder vorbekannt ist.

In der Anamnese mit der Ehefrau ist zu erfahren, dass der Patient eine fortgeschrittene koronare Herzerkrankung (KHK), eine bekannte Lungenfibrose sowie ein Bronchialkarzinom hat. Über eine Therapielimitierung kann die Ehefrau akut jedoch keine Aussage treffen.

Nach weiteren 4–5 min, während bereits der Transport mit Unterstützung der Feuerwehr zur Tragehilfe organisiert wird, wird der Patient mit einer pulslosen elektrischen Aktivität (PEA) wieder reanimationspflichtig und es werden erneut Maßnahmen nach ALS-Algorithmus begonnen.

Inzwischen sind neben der Ehefrau auch die Schwägerin sowie die beiden Kinder des Patienten an der Einsatzstelle eingetroffen und im Gespräch kann nun auch die Frage nach einer Patientenverfügung bzw. dem mutmaßlichen Patientenwillen in Bezug auf medizinische Maßnahmen beantwortet werden. Von den Angehörigen wird unabhängig voneinander bestätigt, dass der Patient aufgrund seiner Vorerkrankungen und der damit verbundenen deutlichen Einschränkung seiner Lebensqualität keine Reanimations- oder Intensivbehandlung gewünscht hätte.

Das Einsatzteam kann während des Gesprächs des Notarztes mit der Familie nach 4 min erneut einen ROSC erreichen. In Anbetracht der nun neuen Informationen erfolgt eine Rücksprache mit dem gesamten Team und es wird im einstimmigen Konsens beschlossen, dass die laufenden lebenserhaltenden Maßnahmen (maschinelle Beatmung, Medikamentengabe) nicht weiter fortgesetzt werden. Zum einen stehen die Maßnahmen im Widerspruch zum Patientenwillen und zum anderen wird auch vom medizinischen Gesichtspunkt her eine sehr eingeschränkte Prognose auf Überleben angenommen.

Nach Diskonnektion des Beatmungsgeräts sowie Beendigung der Medikamentenzufuhr kommt es rasch zu einem Abfall von Herzfrequenz, Sauerstoffsättigung und Blutdruck. Der Patient verstirbt 70 min nach Alarmierung an der Einsatzstelle im Beisein seiner Angehörigen.

### Fallbeispiel 3

Alarmierung des Rettungsdienstes (NEF und RTW) erfolgt um 18.48 Uhr mit dem Einsatzstichwort „Synkope“.

Während der Anfahrt wird mitgeteilt, dass es sich um eine Reanimationssituation bei einem 91-jährigen Patienten handelt, aus psychischen Gründen jedoch keine Reanimation durch die anwesende Person (24-h-Pflegekraft) durchgeführt wird. Bei Eintreffen des NEF um 18.57 Uhr hat die RTW-Besatzung bereits mit der Reanimation begonnen. Da keine Angehörigen anzutreffen sind und keine Patientenverfügung vorliegt, wird die Reanimation fortgesetzt. Trotz initialer Asystolie kann nach 12 min ein ROSC erzielt werden. Im 12-Kanal-EKG zeigt sich ein Sinusrhythmus mit Linksschenkelblock, sodass bei V.a. vorliegenden Myokardinfarkt ein Transport in ein Herzkatheterlabor initiiert wird. Während der Transportvorbereitung erscheint eine Nachbarin des Patienten, die seine Patientenverfügung vorlegt, in der der Patient eine Reanimation ablehnt. Da inzwischen gute Blutdruckwerte sowie ein stabiler Sinusrhythmus ohne Katecholamintherapie vorliegen, wird im Team entschieden, den Transport des Patienten fortzusetzen. Während des Transports wird der Sohn des Patienten, der als Vorsorgebevollmächtigter eingesetzt ist, telefonisch informiert. Dieser bestätigt den Wunsch seines Vaters, nicht reanimiert werden zu wollen.

Im Zielkrankenhaus wird daraufhin von einer Herzkatheteruntersuchung abgesehen. Nach Eintreffen des Sohns wird die Therapie im Krankenhaus auf ein palliatives Konzept umgestellt und der Patient verstirbt kurz nach Mitternacht.

## Hintergrund

Daten des deutschen Reanimationsregisters zeigen, dass das Durchschnittsalter der im Jahr 2023 außerklinisch reanimierten Patienten 69,9 Jahre betrug. Hierbei waren nur 1,4 % jünger als 18 Jahre, jedoch 32,7 % aller Patienten älter als 80 Jahre [1]. Über die letzten Jahre konnte insbesondere in dieser Altersgruppe ein deutlicher Anstieg beobachtet werden, so gehörten im Jahr 2017 lediglich 29,6 % der reanimierten Patienten dieser Altersgruppe an [2].

Bei Aufnahme ins Krankenhaus zeigten im Jahr 2023 32,5 % aller reanimierten Patienten einen ROSC, jedoch wurden nur 10,4 % der außerklinisch reanimierten Patienten lebend aus der Krankenhausbehandlung entlassen [1].

Verschiedene Studien konnten zeigen, dass ein hohes Patientenalter sowohl grundsätzlich mit einem schlechten Outcome bei prähospitalen Reanimationen als auch mit einem schlechten neurologischen Outcome bei Krankenhausentlassung einhergeht [3–5]. Weitere Prädiktoren für ein ungünstiges Outcome sind eine initiale Asystolie sowie schwere Vorerkrankungen [3]. Diesbezüglich wurden auch Entscheidungshilfen, sog. Termination-of-Resuscitation-Regeln (ToR), entwickelt, die die Mitarbeitenden im Rettungsdienst bei der Entscheidung zum Abbruch einer Reanimation unterstützen sollen [3, 6, 7].

Eine schriftlich verfasste Patientenverfügung, durch die der Wille des Patienten für eine konkrete Lebens- und Behandlungssituation eindeutig und sicher festgestellt werden kann, ist für die behandelnden Ärzte seit Inkrafttreten des „Patientenverfügungsgesetzes“ im Jahr 2009 bindend (§ 1901 a Abs. 1 BGB; [8–10]). Auch in der letzten Leitlinie des European Resuscitation Council wird auf die Beachtung von Patientenverfügungen eingegangen (vgl. Infobox 1). Eine gültige und relevante Patientenverfügung, die Reanimationsmaßnahmen verneint, wird in der Leitlinie als Grund zum Nichteinleiten oder als Rechtfertigung für den Abbruch einer Reanimation benannt [11]. Auch wenn in der deutschen Gesellschaft immer noch ein Mangel an Vorsorgedokumenten besteht, konnte in diversen Erhebungen gezeigt werden, dass insbesondere ältere und vorerkrankte Menschen eine Patientenverfügung verfassen [12–18]. In diesen werden gerade lebensverlängernde Maßnahmen in den allermeisten Fällen abgelehnt [19]. Bei einer deutschen Kohorte haben Rupp et al. retrospektiv den Einfluss einer vorliegenden Patientenverfügung auf die Reanimationsdauer untersucht und zeigen können, dass Patienten mit einer Patientenverfügung signifikant älter und schwerer vorerkrankt waren [17]. Paratz et al. konnten in einer retrospektiven Erhebung in Australien zeigen, dass der Anteil der Patienten mit DNR-Status mit dem Alter zunimmt, aber auch, dass trotz DNR-Status circa 4 % der präklinisch reanimierten Patienten in ein Krankenhaus transportiert wurden. In einer Befragung des medizinischen Personals nach dem Grund des Transports wurde hierbei als häufigster Grund genannt, dass bereits ein ROSC erzielt wurde [20].

Neben der nichtgewollten Behandlung, deren Zulässigkeit in den nächsten beiden Abschnitten auch noch unter juristischen und ethischen Aspekten betrachtet wird, kommt es hierdurch auch zu einer Übertherapie und unnötigen Kosten für das Gesundheitssystem. Als Übertherapie wird im Allgemeinen eine Behandlung bezeichnet, die dem Patienten mehr schädigt als nützt und zumeist von wirtschaftlichen Anreizen getriggert ist. Die Übertherapie ist dabei nicht nur für den Patienten schädlich, sondern führt auch zu Zweifeln an der Sinnhaftigkeit des eigenen Tuns bei den Behandlern, wodurch Moral Distress entsteht, die Burn-out-Rate ansteigt und es zur Abwanderung von Fachkräften aus dem Gesundheitssektor kommt [21]. Die Sektion Ethik der Deutschen Interdisziplinären Vereinigung für Intensiv- und Notfallmedizin (DIVI) hat diesbezüglich ein wertvolles Positionspapier verfasst, jedoch bedarf es in der Zukunft sicher auch gesetzlicher Änderungen und anderer Abrechnungsmodalitäten, um das Problem besser zu adressieren [22].

Neben der betroffenen Person und den Behandlern muss man ebenso die Angehörigen betrachten, die einer starken seelischen Belastung ausgesetzt werden. Bei der Fortführung einer aussichtslosen Behandlung befinden sich die Angehörigen in einem Kreislauf aus Hoffen und Bangen und stehen mitunter als Bevollmächtigte vor schweren Entscheidungen, die – anders als in der Intensivmedizin – bei einer Reanimation im häuslichen Umfeld in kürzester Zeit getroffen werden müssen [21].

### Infobox Aussagen der ERC-Leitlinie zum Umgang mit Patientenverfügungen [23]

- Eine Patientenverfügung ist ein Instrument, das Informationen über die Präferenzen und Ziele einer Person in Bezug auf medizinische Verfahren und Behandlungen weitergibt, insbesondere für Entscheidungen am Lebensende.
- Patientenverfügungen sollen die Autonomie des Patienten auf Situationen ausdehnen, in denen er nicht in der Lage ist, seine Präferenzen in Bezug auf Behandlungsentscheidungen auszudrücken. Sie spiegeln die individuellen moralischen, kulturellen und religiösen Einstellungen eines Patienten wider.
- Grundsätzlich müssen Patientenverfügungen die folgenden 3 Kriterien erfüllen: Vorhandensein, Gültigkeit (durch regelmäßige Überprüfung realisiert) und Anwendbarkeit.
- Angehörige von Gesundheitsberufen sollten feststellen, ob ihre Patienten eine Patientenverfügung haben.
- Ärzte sollten die Patientenverfügungen ihrer Patienten respektieren und sie in ihre Entscheidungsfindung einbeziehen.
- Ärzte sollten mit ihren Patienten über Patientenverfügungen sprechen.
- Es sollte versucht werden, die Wünsche des Patienten (insbesondere von Patienten mit unheilbaren Krankheiten) in Bezug auf lebenserhaltende Behandlungen zu ermitteln, wenn der Patient entscheidungsfähig ist, oder alternativ von seinen Vertretern, wenn der Patient nicht entscheidungsfähig ist.
- Wenn sich Patientenverfügungen auf die Ablehnung einer bestimmten Behandlung beziehen, sollte sorgfältig geprüft werden, ob dies auch für ähnliche (aber immer noch alternative) Behandlungen gelten sollte oder nicht.
- Alte (z. B. > 5 Jahre) und nichtaktualisierte Patientenverfügungen sollten im Zusammenhang mit der Verfügbarkeit neuer, sichererer und potenziell wirksamerer Therapien vorsichtig interpretiert werden.
- Nichtstandardisierte Verfügungen (z. B. Tätowierungen, die anzeigen, dass keine Herz-Lungen-Wiederbelebung versucht werden soll – DNACPR) sollten nicht sofort als rechtsgültig angesehen werden, es sei denn, dies ist durch das örtliche Recht so festgelegt.
- In Ländern, in denen nichtstandardisierte Patientenverfügungen als rechtsgültig angesehen werden, kann die Durchführung einer Reanimation unter Bedingungen, bei denen eine Wiederbelebung wahrscheinlich erfolglos ist, zu einer strafrechtlichen Verfolgung des medizinischen Fachpersonals führen. Gleichzeitig sollten umgehend alle Anstrengungen unternommen werden, um zu klären, ob eine gültige, einschlägige Patientenverfügung vorliegt.
- Patientenverfügungen sollten nicht befolgt werden, wenn:
  - die Patientenverfügung eine Handlung fordert, die durch die Gesetze und/oder Vorschriften des Lands verboten ist;
  - es überzeugende Beweise dafür gibt, dass der Patient seine Meinung seit dem Ausfüllen der Patientenverfügung geändert hat;
  - es überzeugende Beweise dafür gibt, dass der Patient die Art der von ihm ausgefüllten Patientenverfügung nicht verstanden hat;
  - es Beweise dafür gibt, dass der Patient zum Zeitpunkt der Erstellung der Verfügung keine freie Wahl hatte.

## Juristische Aspekte

„Die medizinische Indikation begrenzt den Inhalt des ärztlichen Heilauftrags“ (vgl. BGH JZ 2003, 732, 737). Damit kann die Durchführung einer lebensrettenden oder -verlängernden Maßnahme durch den Arzt verweigert werden, wenn die medizinische Indikation dafür nicht (mehr) vorliegt. Die Indikation für eine Heilbehandlung kann nur von Ärzten gestellt werden und beruht auf den Grundsätzen der evidenzbasierten Medizin sowie der persönlichen Expertise und dem Wissen um Krankheitsverläufe und die individuelle Prognose. Hierbei ist von immanenter Bedeutung, dass eine medizinische Sinnhaftigkeit des Heileingriffs besteht und ebenso ein erreichbares Therapieziel existiert. Sofern kein Therapieziel formuliert werden kann, verbietet sich die Behandlung, da dann nur ein Schaden für den Patienten resultiert, ohne konkreten Nutzen für die betroffene Person.

**Abb. 1:**
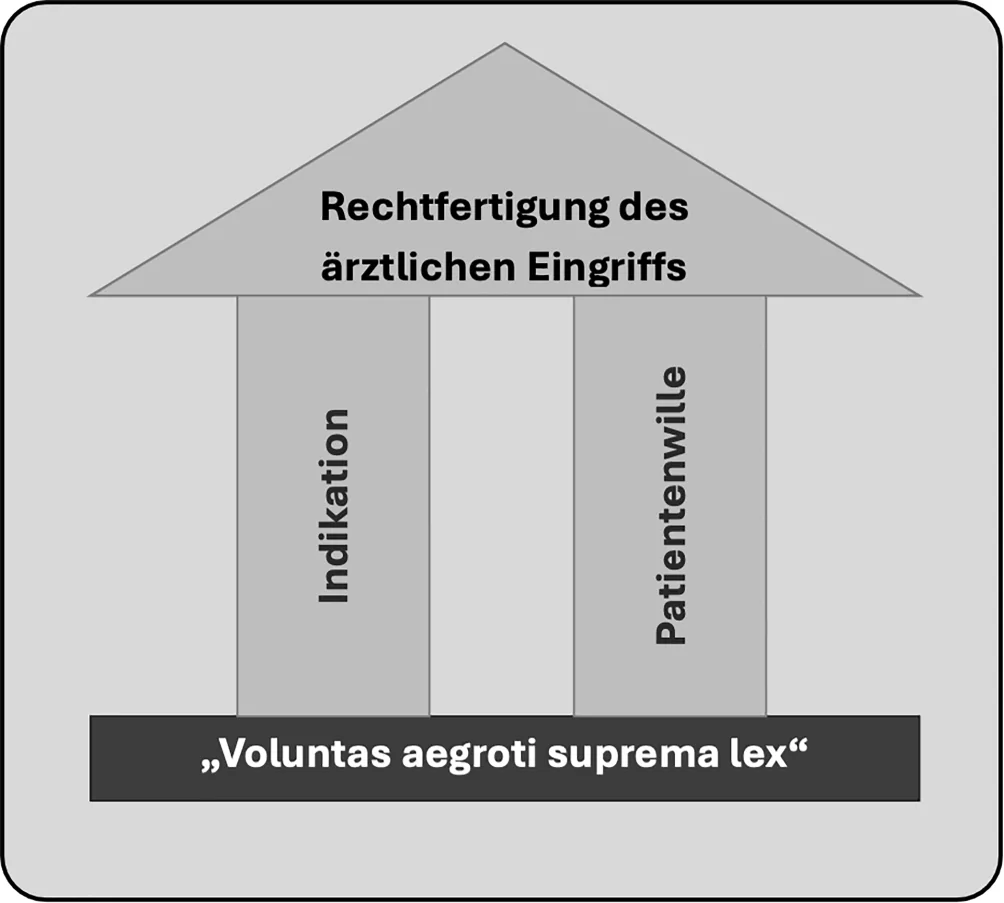
Zwei-Säulen-Modell

Entsprechend des Grundgesetztes ist jeder „grundsätzlich frei, über Eingriffe in seine körperliche Integrität und den Umgang mit seiner Gesundheit nach eigenem Ermessen zu entscheiden“ [24, 25]. Nach ständiger Rechtsprechung erfüllt jeder Eingriff in die körperliche Unversehrtheit – und damit auch ein medizinisch indizierter Heileingriff – den objektiven Tatbestand der Körperverletzung im Sinne der §§ 223 ff StGB [26]. Solche Eingriffe können aber durch ausdrückliche oder mutmaßliche Einwilligung des Patienten gerechtfertigt werden. Die Rechtfertigung für eine medizinische Behandlung ist somit nur gegeben, wenn erstens die Indikation für den Eingriff besteht und zweitens die Einwilligung des Patienten gegeben ist (sog. „Zwei-Säulen-Modell“, vgl. Abb. 1; [9]).

Nur durch umfassende Aufklärung und Darlegung aller Behandlungsoptionen kann der Patient in die Lage versetzt werden, über die Durchführung der medizinischen Maßnahme zu entscheiden und eine informierte Einwilligung abzugeben (engl. „informed consent“) oder aber die medizinische Maßnahme abzulehnen [27].

**Abb. 2:**
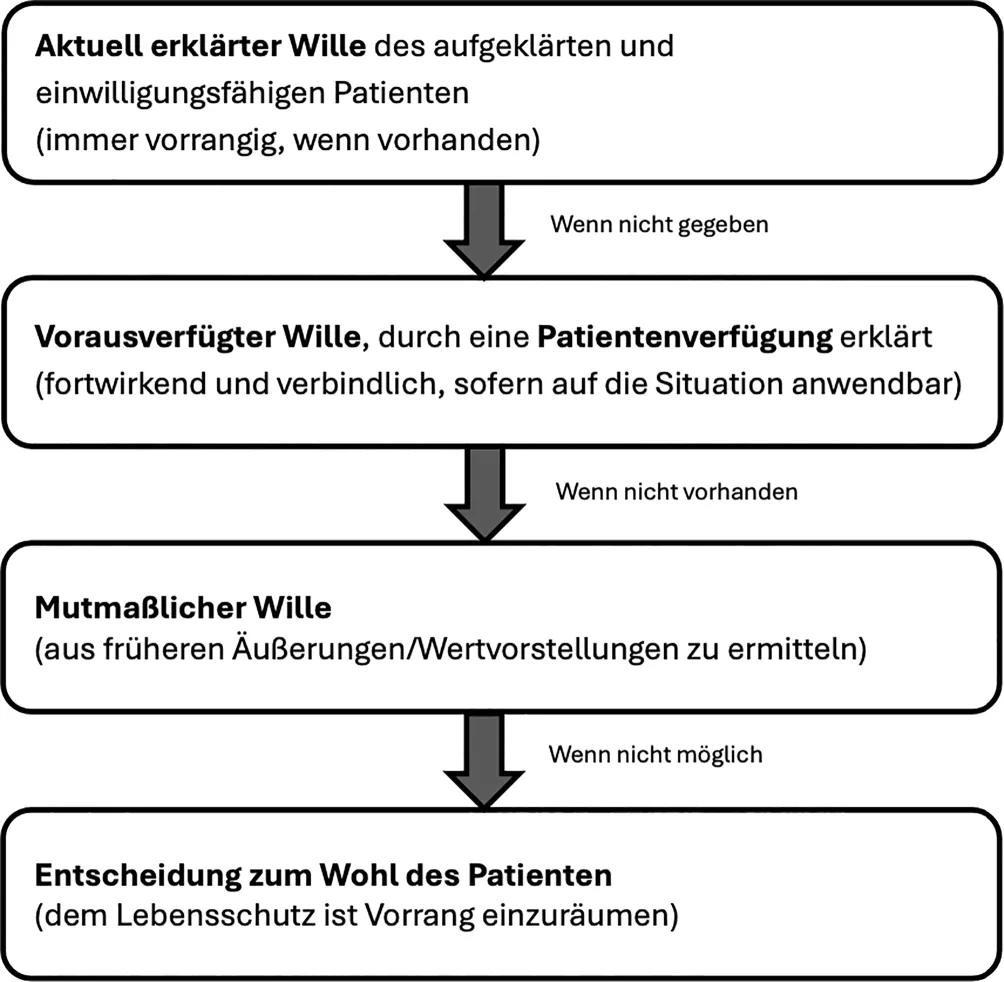
Ermittlung des Patientenwillen

Behandeln Ärzte bewusstlose Patienten in Notfallsituationen, kann naturgemäß keine ausdrückliche Einwilligung durch die betroffene Person gegeben werden. Da ein Patient auch direkt nach ROSC in der Regel nicht selbst die Einwilligung geben kann, tritt an dieser Stelle entweder ein vorausverfügter Wille, beispielsweise in Form einer Patientenverfügung, oder, falls diese nicht vorliegt oder bekannt ist, der hilfsweise herangezogene mutmaßliche Wille des Patienten in Kraft. Beim mutmaßlichen Willen wird durch in der Vergangenheit getätigte Äußerungen, persönliche Überzeugungen etc. ermittelt, ob die betroffene Person der akut notwendigen Heilbehandlung zustimmen oder diese ablehnen würde. Hierbei bedarf es des Einbezugs von Angehörigen oder engen Bekannten, die meist entsprechende Informationen liefern können. Sollte dies nicht möglich sein, wird der Patient entsprechend der medizinischen Indikation mit Ziel des Erhalts des Lebens unter der Prämisse „in dubio pro vita“ (deutsch: „Im Zweifel für das Leben“) behandelt, da grundsätzlich von einer mutmaßlichen Einwilligung des Betroffenen zur Rettung des Lebens ausgegangen wird. Dennoch gilt es, wenn die Notfallsituation behoben ist, den Patientenwillen zu ermitteln und die weitere Behandlung danach auszurichten ([8, 9]; vgl. Abb. 2).

Sowohl die medizinische Indikation als auch die Einwilligung in den Eingriff stehen im konkreten Fall der möglichen palliativen Extubation nach ROSC zur Disposition. Allerdings darf es durch die Überprüfung von Indikation und Patientenwille nicht zu einer unverantwortbaren Verzögerung von lebensrettenden Maßnahmen kommen [27].

Auf der einen Seite kann die medizinische Indikation entfallen, wenn das angestrebte Behandlungsziel unerreichbar geworden ist oder eine realistische Nutzen-Risiko-Abwägung derart negativ ausfällt, dass es medizinisch nicht vertretbar ist, den Heileingriff durch- oder fortzuführen (Schaden > Nutzen; [28]).

Die ärztliche Professionalität gebietet es hierbei, die geäußerten Vertretbarkeitsgrenzen keinesfalls zu überschreiten, selbst bei äußeren Einwirkungsversuchen z. B. durch Familienangehörige [28]. Dies bedeutet, dass sich keine Behandlung „gewünscht“ werden kann, sofern keine Indikation vorliegt (z. B. Fortführung einer Reanimation bei festgestellter infauster Prognose).

Auf der anderen Seite und womöglich weit schwieriger eindeutig zu erfassen liegt die Einwilligung des Patienten in einen potenziellen Heileingriff, in unseren Fallbeispielen in die Fortführung der akuten medizinischen Maßnahmen nach ROSC, ohne die vermutlich ein erneuter Kreislaufstillstand und damit der Tod des Patienten einhergeht. Die Einwilligung stellt die Entscheidung des Patienten dar, ob das medizinisch Machbare auch mit seinen persönlichen Werten und Vorstellungen in Einklang zu bringen ist [27].

Der Behandlungsabbruch – früher auch passive Sterbehilfe genannt – bezeichnet die Beendigung lebensverlängernder Maßnahmen. Geht er auf den Willen des Patienten zurück, ist er nicht strafbar. Vielmehr darf auch eine lebenserhaltende Maßnahme nicht eingeleitet oder fortgeführt werden, sofern der Wunsch nach Behandlungsabbruch gegeben ist. Ein Verstoß dagegen kann nach geltendem Recht eine strafbare Körperverletzung darstellen.

Patientenverfügungen bedürfen jedoch einer gewissen Form. Sie müssen schriftlich vorliegen und mindestens eigenhändig vom Patienten unterschrieben sein (vgl. § 126 BGB). Für den Notfall haben sich Kurzfassungen bewährt, die z. B. in Form einer Ampel oder eines Ausweises die relevanten Wünsche des Patienten übersichtlich darstellen [29]. Insbesondere für die sehr umfassende Form einer Patientenverfügung durch Advance Care Planning (ACP) ist der Notfallbogen als Zusammenfassung hilfreich, um dem behandelnden Rettungsteam eine schnelle Übersicht über medizinisch nicht indizierte sowie individuell nicht gewünschte Behandlungsmaßnahmen zu geben [30].

Außerdem muss durch den behandelnden Arzt und den Vorsorgebevollmächtigten, bzw. den gesetzlichen Betreuer, geprüft werden, ob die vorliegende Verfügung auch für die konkret vorliegende Lebens- bzw. Behandlungssituation zutreffend ist [9, 25].

Ist dies nicht der Fall, tritt nun an die Stelle der Patientenverfügung der mutmaßliche Wille des Patienten. Falls es einen gesetzlichen Betreuer oder einen Vorsorgebevollmächtigen gibt, ist der mutmaßliche Wille durch diesen zu ermitteln und gegenüber dem Behandler zu vertreten (vgl. § 1827 BGB). Der behandelnde Arzt soll mit dem Betreuer bzw. Vorsorgebevollmächtigten zusammen den Patientenwillen im Gespräch eruieren (§ 1828 BGB). Der mutmaßliche Patientenwille ist dabei nach konkreten Anhaltspunkten zu ermitteln [25, 31].

Dazu können Patientenverfügungen, auch wenn diese nicht auf die konkrete Situation zutreffen, herangezogen werden oder auch mündliche Äußerungen, die der Patient zuvor gegenüber Angehörigen o. ä. gemacht hat, berücksichtigt werden [9, 25]. In akuten Notfallsituationen obliegt es jedoch im Zweifel dem behandelnden Arzt, den Patientenwillen zu ermitteln [27].

Eine Therapiezieländerung kann auch nach erreichtem ROSC im Rahmen einer Reanimation im Rettungsdienst durch neue, zusätzliche Informationen im Verlauf des Einsatzgeschehens angezeigt sein. Zum einen kann die medizinische Indikation zur Fortführung der Maßnahmen nicht mehr gegeben sein, beispielsweise wenn aufgrund einer ausgesprochen langen No-flow-Zeit die Prognose katastrophal ist oder die medizinische Vorgeschichte in Zusammenschau mit der aktuellen Situation es nicht mehr zulässt, das angestrebte Therapieziel (z. B. Autonomie oder Teilhabe) zu erreichen.

Auf der anderen Seite kann die Einwilligung des Patienten in den Heileingriff fehlen, entweder durch einen konkreten, vorausverfügten Willen oder nach Ermittlung des mutmaßlichen Willens mit Bevollmächtigen, Ehe- oder Lebenspartnern sowie Angehörigen.

In diesen Situationen kann es medizinisch erforderlich und juristisch geboten sein, das Therapieziel mit klarer Indikation hinsichtlich einer Palliation mit Best Supportive Care zu ändern und alle weiteren Maßnahmen an diesem neuen Therapieziel auszurichten.

## Ethische Aspekte

In ihrem Werk „Principles of Biomedical Ethics“ beschreiben Beauchamp und Childress die Patientenautonomie, neben der Schadensvermeidung, der Fürsorge und der Gerechtigkeit, als eines der 4 Grundprinzipien biomedizinischer Ethik [32]. Die philosophische Grundlage der Autonomie, wie sie von den Philosophen Immanuel Kant (1724–1804) und John Stuart Mill (1806–1873) interpretiert und als ethischer Grundsatz akzeptiert wurde, besteht darin, dass Menschen einen intrinsischen und unbedingten Wert besitzen und daher die Macht haben sollten, rationale Entscheidungen und moralische Wahlmöglichkeiten zu treffen, und dass es jedem gestattet sein sollte, seine Fähigkeit zur Selbstbestimmung auszuüben [33]. Dies bedeutet für die medizinische Praxis, dass Patienten selbstständig und informiert medizinische Entscheidungen treffen können, sei es in Form der direkten Kommunikation mit dem behandelnden Arzt, einer Patientenverfügung, eines Betreuers, eines Bevollmächtigten oder über Angehörige. Die wichtigsten Voraussetzungen zur Durchführung jeder ärztlichen Maßnahme sind die medizinische Indikation und die Einwilligung des Patienten (vgl. Abb. 1; [9]). Bei Entscheidungen am Lebensende kann es jedoch zu Konflikten zwischen den Grundprinzipien der Medizinethik kommen, insbesondere zwischen Autonomie und Fürsorge. Das Recht des Patienten auf Selbstbestimmung trifft auf das Bedürfnis des behandelnden Teams, Fürsorge zu leisten [34, 35]. Schon 1988 befassten sich Pellegrino und Thomasma mit derlei Konflikten und kamen zu dem Schluss, dass die Fürsorge die Autonomie einschließen kann, da die besten Interessen der Patienten eng mit deren Präferenzen verknüpft sind, woraus die primären Pflichten der Ärzte ihnen gegenüber abgeleitet werden [36]. Auch in den aktuellen ethischen Empfehlungen zur „Ethik der Reanimation und Entscheidungen am Lebensende“ der Leitlinie des ERC findet sich das Prinzip der Autonomie wieder. Es wird empfohlen, die Autonomie durch eine gemeinsame Entscheidungsfindung und, wenn diese nicht möglich ist, durch Patientenverfügungen und vorausschauende Behandlungsplanung umzusetzen [37]. In Notfallsituationen kann es jedoch dazu kommen, dass der Wille des Patienten nicht bekannt ist und auch nicht unmittelbar ermittelt werden kann. Dann ist eine medizinisch indizierte Behandlung einzuleiten, die die Erhaltung des Lebens zum Ziel hat. Im weiteren Verlauf muss die Situation dann erneut beurteilt und festgestellt werden, ob die eingeleiteten Maßnahmen weiterhin indiziert und vom Patientenwillen getragen sind [38, 39]. Es wird in der Leitlinie jedoch nicht explizit erwähnt, wie mit Patienten umgegangen werden soll, bei denen sich nach Wiedererlangen des Kreislaufs herausstellt, dass eine Reanimation und die damit einhergehenden Maßnahmen nicht vom Patienten gewollt gewesen wären. Aus den „Empfehlungen der Bundesärztekammer und der Zentralen Ethikkommission bei der Bundesärztekammer zum Umgang mit Vorsorgevollmacht und Patientenverfügung in der ärztlichen Praxis“ geht eindeutig hervor, dass eine medizinische Maßnahme im Einklang mit dem Willen des Patienten unterlassen werden muss, wenn es Einigkeit darüber gibt, dass der Patient diese Maßnahme ablehnen würde [38]. Dies gilt für jegliche Maßnahmen, auch solche, die eindeutig medizinisch indiziert sind, somit also auch für die maschinelle Beatmung bei einem Patienten mit ROSC. Einzig wenn Zweifel am Willen des Patienten bestehen und bei Unterlassen der Maßnahme der Tod oder schwerer, lang andauernder Schaden entstünde, kann die Maßnahme bis zur Klärung weitergeführt und das Betreuungsgericht eingeschaltet werden [38, 39]. Wenn man die Prinzipien der biomedizinischen Ethik berücksichtigt, ist es also unmissverständlich angezeigt, Patienten mit einem ROSC in der Postreanimationsphase zu extubieren, wenn aus einer gültigen Patientenverfügung oder durch Bevollmächtigte, Betreuer oder Angehörige eindeutig der Wille des Patienten ermittelt werden kann und dieser nicht im Einklang mit den durchgeführten oder zukünftig notwendigen Maßnahmen steht. Eine Weiterführung der Maßnahmen würde nicht nur die Autonomie des Patienten verletzen, sondern wäre nicht zum Wohle des Patienten und würde ihm, durch eine Entmündigung, im Zweifel sogar schaden. Für die praktische Umsetzung empfiehlt das Positionspapier der Sektion Ethik der DIVI ein „ethical team time out“, um eine multiprofessionelle Teamentscheidung herbeizuführen. Dabei soll zum einen geprüft werden, ob die medizinische Maßnahme weiterhin indiziert ist und im zweiten Begutachtungsschritt auch vom Patienten gewünscht ist. Durch diese Form der partizipativen Entscheidungsfindung soll auch über die aktuelle Situation hinausgedacht werden und eine transparente und für alle Beteiligten tragbare Lösung gefunden werden [40].

## Handlungsempfehlung

**Abb. 3:**
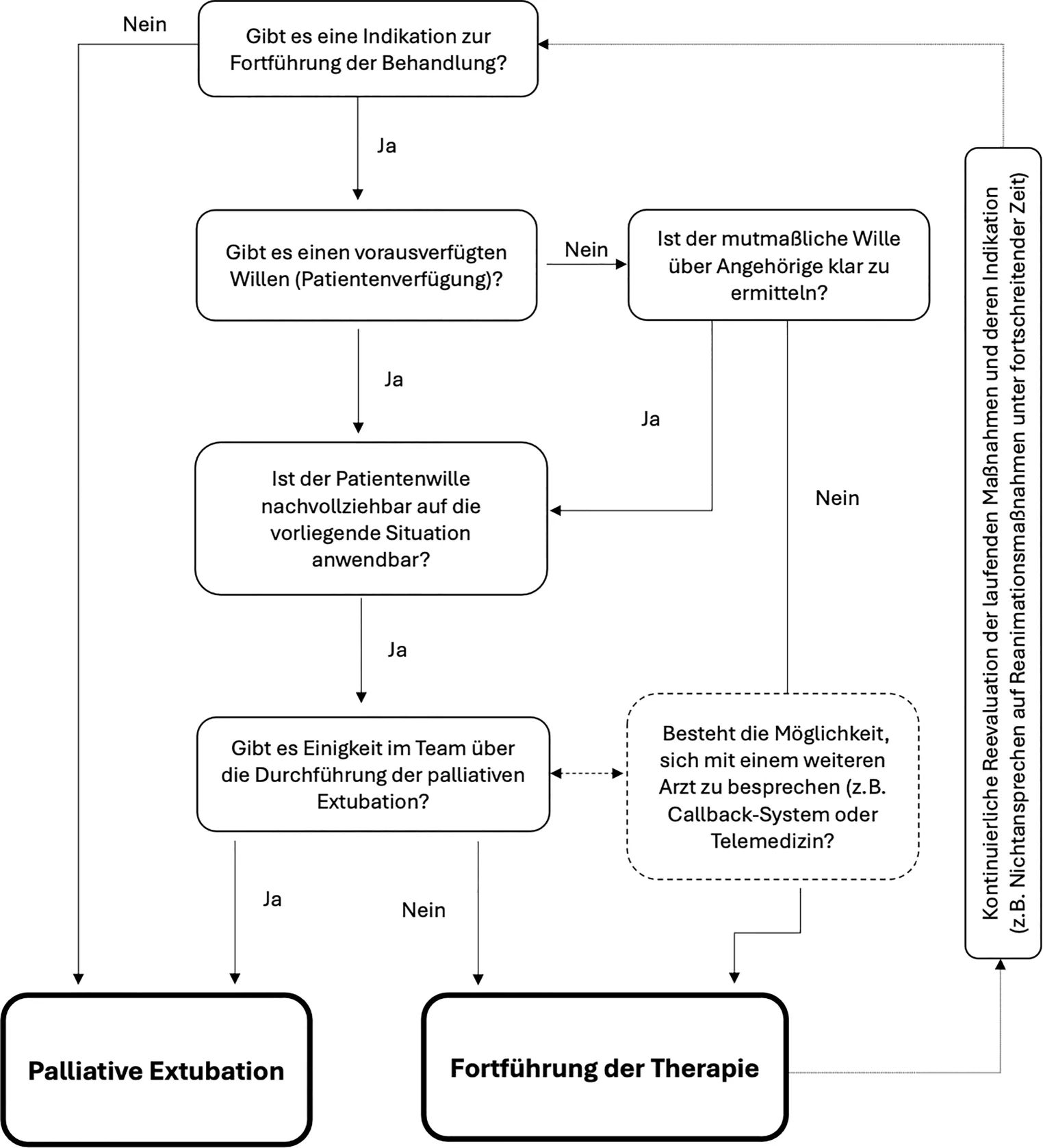
Flussdiagramm zur Entscheidungsfindung

In Abb. 3 sind relevante Punkte für die Entscheidungsfindung zur Fortführung der Behandlung bzw. zur Durchführung einer palliativen Extubation dargestellt.

Im klinischen Alltag werden Entscheidungen über Therapien und insbesondere über deren Beendigung in der Regel nicht von Ärzten in der Facharztausbildung allein, sondern in Absprache mit den zuständigen Fachärzten getroffen. Die Zusatzbezeichnung „Notfallmedizin“ darf in Deutschland bereits nach 2 Jahren klinischer Tätigkeit erworben werden. Im präklinischen Bereich werden daher auch von Nichtfachärzten eigenständige Entscheidungen erwartet, zu denen sie als approbierte Ärzte auch legitimiert sind. Die Untersuchung von Heuser et al. hat in diesem Zusammenhang gezeigt, dass Assistenzärzte in der Präklinik signifikant länger reanimieren als Fachärzte [5]. Es kann also davon ausgegangen werden, dass eine Therapiebeendigung, wie die von uns beschriebene palliative Extubation, Berufsanfängern schwerer fällt als Fachärzten. Wie in Abb. 3 dargestellt, kann es somit hilfreich sein, ein Callback- oder Telenotarztsystem vorzuhalten, um sich mit einem weiteren Notarzt in Bezug auf die Maßnahme abzusprechen. Diese Möglichkeit bietet nicht nur Assistenzärzten, sondern auch Fachärzten und allen anderen Mitgliedern des Rettungsteams zusätzliche Sicherheit und einen erweiterten Blick auf die Situation [41].

Wenn es im Team und mit den Stellvertretern des Patienten Einigkeit über die Beendigung der Behandlungsmaßnahmen gibt und eine palliative Extubation umgesetzt werden soll, empfiehlt es sich, den Patienten mit einem Opioid (z. B. Morphin oder Fentanyl) gegen potenziell auftretende Dyspnoe abzuschirmen. Zusätzlich kann auch der Einsatz eines Benzodiazepins (z. B. Midazolam) erwogen werden. Die Gabe von kreislaufunterstützenden Medikamenten (z. B. Adrenalin) sollte zunächst beendet werden, bevor im nächsten Schritt die Beatmung eingestellt wird. Hierbei kommen 2 Vorgehensweisen in Betracht, das *Compassionate Weaning* oder die *Compassionate Extubation*. Beim *Compassionate Weaning* wird der Beatmungsweg belassen und nur die Beatmungsunterstützung reduziert, während bei der *Compassionate Extubation* die Beatmungstherapie beendet und die Atemwegssicherung entfernt wird. Für beide Varianten gibt es Vor- und Nachteile, die im Konsensuspapier „Palliativmedizinische Aspekte in der klinischen Akut- und Notfallmedizin sowie Intensivmedizin“ unter Punkt 6 dargestellt sind [42].

## Fazit für die Praxis

- Dass man als Notarzt in die Situation kommt, über eine palliative Extubation nach primär erfolgreicher Reanimation nachdenken zu müssen, ist eine seltene aber durchaus mögliche Gegebenheit. Es gibt dabei sowohl eine juristische wie auch ethische Betrachtungsweise und es bedarf guter Kenntnis über die Hintergründe, die in diesem Übersichtsartikel vorgestellt wurden.
- Wenn der Patientenwille einwandfrei evaluiert werden kann und die Indikation zur Fortführung der Behandlung nicht gegeben ist, muss die Extubation durchgeführt werden und der Patient im Sterben symptomorientiert begleitet werden.
- Sollte es hingegen Zweifel an der Zulässigkeit des Patientenwillen geben, ist die weitere Behandlung angezeigt und die Entscheidung sollte auf einen späteren Zeitpunkt verschoben werden. Wenn der Gesundheitszustand stabilisiert ist und man ausreichend Zeit hatte alle Informationen zusammenzutragen, kann gegebenenfalls mithilfe einer Ethikfallberatung und dem Einbezug der nächsten Angehörigen eine Strategie für die weitere Behandlung erarbeiten kann.
